# *Propionibacterium avidum* as an Etiological Agent of Prosthetic Hip Joint Infection

**DOI:** 10.1371/journal.pone.0158164

**Published:** 2016-06-29

**Authors:** Peter Wildeman, Holger Brüggemann, Christian F. P. Scholz, Andreas Leimbach, Bo Söderquist

**Affiliations:** 1 Department of Orthopedics, Faculty of Medicine and Health, Örebro University, Örebro, Sweden; 2 Department of Biomedicine, Aarhus University, Aarhus, Denmark; 3 Institute of Hygiene, University of Münster, Münster, Germany; 4 School of Medical Sciences, Faculty of Medicine and Health, Örebro University, Örebro, Sweden; University of Ulster, UNITED KINGDOM

## Abstract

*Propionibacterium acnes* is well-established as a possible etiologic agent of prosthetic joint infections (PJIs). Other *Propionibacterium* spp. have occasionally been described as a cause of PJIs, but this has not previously been the case for *P*. *avidum* despite its capacity to form biofilm. We describe two patients with prosthetic hip joint infections caused by *P*. *avidum*. Both patients were primarily operated with an anteriorly curved skin incision close to the skin crease of the groin, and both were obese. Initial treatment was performed according to the DAIR procedure (debridement, antibiotics, and implant retention). In case 1, the outcome was successful, but in case 2, a loosening of the cup was present 18 months post debridement. The *P*. *avidum* isolate from case 1 and two isolates from case 2 (obtained 18 months apart) were selected for whole genome sequencing. The genome of *P*. *avidum* obtained from case 1 was approximately 60 kb larger than the genomes of the two isolates of case 2. These latter isolates were clonal with the exception of SNPs in the genome. All three strains possessed the gene cluster encoding exopolysaccharide synthesis.

*P*. *avidum* has a pathogenic potential and the ability to cause clinically relevant infections, including abscess formation, in the presence of foreign bodies such as prosthetic joint components. Skin incision in close proximity to the groin or deep skin crease, such as the anteriorly curved skin incision approach, might pose a risk of PJIs by *P*. *avidum*, especially in obese patients.

## Introduction

A modern technology to replace worn-out joints with artificial implants has significantly improved the quality of life for many people, and is one of the major technical progresses in medicine during the 1900s [1]. In the United States 300,000 patients are operated annually and more than 1 million worldwide. Although total hip replacement is considered a safe procedure, there are a few major complications that may occur in relation to the surgery, and prosthetic joint infection is one of the most feared, with significant costs in both monetary and personal terms. The outcome after a prosthetic joint infection depends on several factors; one factor of major importance is which type of bacteria causes the infection [2].

*Propionibacterium* spp. are slow growing Gram-positive anaerobic bacteria. They are usually rod-shaped and pleomorphic, and are regarded as skin commensals with low or no pathogenic potential. However, *P*. *acnes* in particular has the ability to act as an opportunistic pathogen [3] and several reports indicate that this species may also be the etiological agent of severe infections such as infective endocarditis, bone and joint infections, and infections of the central nervous system, predominantly when foreign bodies are present [4]. Infections of orthopedic prosthetic devices and other implants are significant medical problems [5]. These infections related to foreign materials are generally difficult to treat, since the bacteria associated with the infections produce biofilm that reduces the penetration of antibiotics and counteracts the human immune defense mechanisms [6]. Furthermore, the clinical presentation of infections caused by low-virulence microorganisms is often insidious and gradual, resulting in symptoms and signs of infection several months after surgery.

*P*. *acnes* is now well-established as a possible etiologic agent of prosthetic joint infections (PJIs) [7,8], especially prosthetic shoulder joint infections [9]. Other *Propionibacterium* spp., such as *Propionibacterium granulosum*, has also occasionally been described as a cause of PJIs [10]. *Propionibacterium avidum* has been reported as a cause of soft tissue infections, such as various abscesses [11–13] and of bone and joint infections in at least three cases [14], but has not previously been associated with PJIs. Data regarding *P*. *avidum* is scarce. First analysis of a *P*. *avidum* genome has revealed the presence of an exopolysaccharide (EPS) biosynthesis gene cluster and electron and atomic force microscopy studies have confirmed the presence of an EPS-like structure surrounding cells of *P*. *avidum* [15].

We here report two patients with prosthetic hip joint infections caused by *P*. *avidum*, and present genomic insight in this largely unexplored species.

### Case 1

An 84-year-old woman with a history of seropositive rheumatoid arthritis, obstructive lung disease, and obesity (BMI 38) underwent bilateral cemented total hip arthroplasty due to coxarthrosis in 2009 for the left hip and in 2011 for the right hip. The surgery of the left hip was performed with the Moore (dorsolateral) approach, and functional outcome was good. Current medication was low dose oral and inhaled corticosteroids. In December 2013, she had an accidental fall and dislocated her left hip; this was followed by recurrent dislocations of the left hip replacement. Closed reduction was performed a total of six times.

**Fig 1:**
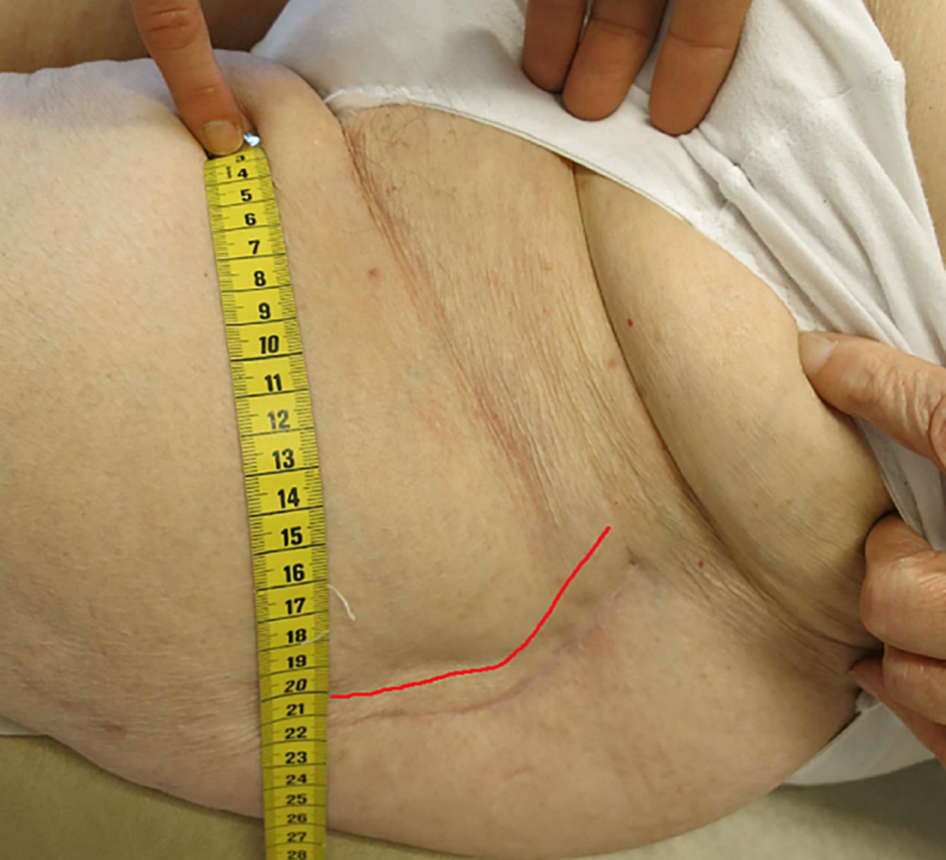
Anteriorly curved skin incision approach. Case 1 left hip and. Red line indicates the anterior skin incision.

In May 2014 she was admitted to the hospital because of dislocation, and a decision was made to perform revision surgery of the left hip. A cup revision was performed with a cemented constrained tripolar cup, using a muscle-sparing Watson-Jones approach [16,17] with an anteriorly curved skin incision (Fig 1). Chlorhexidine was used as skin antisepsis and cloxacillin 2g*3 (-30 min, 2h and 6h) as antibiotic prophylaxis. Perioperative bleeding totaled 1165 mL of blood, and the operation time was 98 minutes. The patient received blood transfusions, but was feeling well postoperatively. The wound did not show any signs of infection at discharge from hospital 8 days after surgery.

Approximately 3 weeks after surgery the patient was readmitted to the hospital with acute onset of redness and intense pain from the hip. Her C-reactive protein (CRP) was 155 mg/L and her leukocyte count was elevated (16.3 x 10^9^), but no fever was present. She received a total of three doses of cloxacillin (2 g each) intravenously. Ultrasonography indicated abscess formation anterior to the left hip. Cloxacillin was discontinued, and an incision and thorough soft tissue debridement including arthrotomy and synovectomy was performed the following day. Pus was detected, and fatty necrosis was excised. The wound was aggressively irrigated and closed over two drains. The prosthesis was assessed as stable. A total of two aspirations of synovial fluid and five tissue biopsies were collected. The samples were cultured on Difco GC Medium Base (BD Diagnos- tic Systems) supplemented with 1% haemoglobin powder, 10% horse serum (SVA, Uppsala, Sweden) and 1% IsoVitalex Enrichment (BD Diagnostic Systems) at 36°C under 5% CO2 for 2 days and on anaerobe agar medium (Fastidious anaerobe agar (Acumedia; Neogen Corporation, Lansing, MI, USA) supplemented with 5% horse blood (SVA)) at 36°C anaerobically for up to 5 days, as well as in enrichment broth (29.7% fastidious anaerobic broth (Lab M, Bury, UK) supplemented with 10% D-glucose (J. T. Baker, Deventer, The Nether- lands)) for 7 days. Cloxacillin was reinstituted, but was discontinued when all cultures showed solely growth of *P*. *avidum*. Species identification was performed by Matrix-assisted laser desorption/ionization time-of-flight mass spectrometry (MALDI-TOF MS) (Microflex LT and Biotyper 3.1, Bruker Daltonics, Bremen, Germany). The antimicrobial susceptibility pattern is shown in Table 1. Treatment was changed to orally administered clindamycin 300 mg three times per day and rifampicin 600 mg once per day. At follow-up approximately 4 weeks after debridement, the patient’s CRP was 26 mg/L and the wound had healed with no signs of infection. Treatment was changed to phenoxymethylpenicillin, administered orally 2 g three times per day for 2 months. CRP was normalized at 1.9 mg/L at a control visit 2 months after debridement. The total treatment time was 3 months. Follow-up for 1 y has been uneventful.

**Table 1:** Antibiotic susceptibility.

Antibiotics (MIC mg/L)	14T	13T	15T
** Penicillin G**	0.047	0.064	0.064
**Oxacillin**	0.75	0.75	0.75
** Ampicillin**	0.047	0.125	0.125
** Clindamycin**	0.032	0.016	0.032
**Metronidazole**	>256	>256	>256
** Ciprofloxacin**	0.094	0.25	0.25
** Moxifloxacin**	0.047	0.125	0.125
** Rifampicin**	0.003	<0.002	<0.002
**TMP-SZ**	5	5	5

Patterns as MIC values determined by Etest of *Propionibacterium avidum* obtained from two patients with prosthetic hip joint infections: one isolate (14T) from case 1, and two isolates (13T and 15T) from case 2 with 18 months between cultures.

### Case 2

**Fig 2:**
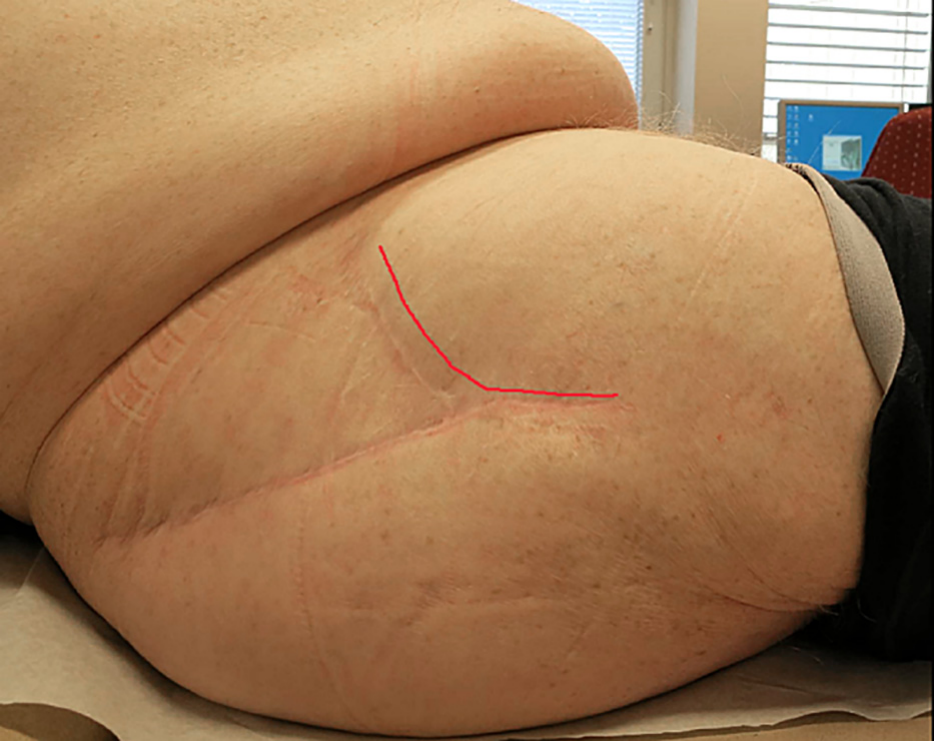
Anteriorly curved skin incision approach. Case 2 right hip. Red line indicates the anterior skin incision.

A 75-year-old man with hypertension and obesity (BMI 37) received a cemented cup and uncemented stem hip prosthesis due to femoral head necrosis in April 2013. The surgery was performed with a muscle-sparing Watson-Jones approach using an anteriorly curved skin incision (Fig 2). He had a Paprosky 2B [18] acetabular defect, and a bone autograft was transplanted as support for the superior rim of the acetabulum. Chlorhexidine was used as skin antisepsis and cloaxcillin 2g*3 (-30 min, 2h and 6h) as antibiotic prophylaxis. Perioperative bleeding totaled 810 mL of blood, and the operating time was 90 minutes. The wound did not show any signs of infection at discharge from hospital 5 days after surgery.

Approximately 3 weeks after surgery, the patient was readmitted to the hospital with a wound showing secretion and redness. His CRP was 64 mg/L, but no fever was present. A superficial wound culture showed growth of coagulase-negative staphylococci, alpha-hemolytic streptococci, and *Propionibacterium* spp. 4 days later incision, thorough soft tissue debridement including arthrotomy, synovectomy and aggressive irrigation. There was a newly formed sinus tract that was excised together with the infected tissue. Six biopsies and an aspiration of synovial fluid were taken; all showed growth of *Propionibacterium* spp., later determined to species level as *P*. *avidum* by MALDI-TOF MS. The antimicrobial susceptibility pattern is shown in Table 1.

The patient was treated intravenously with clindamycin 600 mg three times per day until discharge from hospital 1 week later. At discharge, treatment was changed to oral administration of clindamycin 300 mg three times per day for 5 weeks. Follow-up 4 months postoperatively including X-ray was uneventful, but no blood samples were examined.

However, 18 months after debridement the patient was referred to the primary hospital because of a sudden increase of pain and discomfort in the hip. Plain X-ray revealed loosening of the cup.

The patient was then referred to the university hospital for hip revision surgery. At the preoperative visit, he had severe pain in the hip and CRP of 180 mg/L. Septic loosening was suspected. Aspiration from the hip was performed, and culture showed growth of *P*. *avidum*. Leukocyte cell count from synovial fluid was 47.2 10^9^/L (93% poly vs. 7% mono). A CT scan showed abscess formation below the acetabular floor inside the pelvis, and a Paprosky 3A acetabular defect. The stem was well fixed. The hip prosthesis was extracted the following day in a process which included draining of the abscess, thorough soft tissue debridement, and locally administered gentamicin. This was the first stage in a two-stage hip revision surgery approach for the infected hip arthroplasty. A total of one aspiration and seven tissue biopsies were collected. Treatment was initiated with piperacillin/tazobactam (4 g/0.5 g) three times daily. All cultures showed growth of *P*. *avidum* as before, and the antimicrobial susceptibility pattern was almost identical to the previous findings Table 1. After 8 days, treatment was changed to benzyl-penicillin 1g intravenously three times daily.

After 3 weeks, the patient experienced increased hip pain, and a CT scan showed multiple abscesses around the Girdlestone hip. Another reoperation with debridement, locally administered gentamicin, and drainage was performed. A total of one aspiration and ten biopsies were collected; these showed growth of *P*. *avidum* (n = 4), *Staphylococcus epidermidis* (n = 7; two different antibiotic susceptibility patterns), and *Enterococcus faecalis* (n = 1). Treatment was initially piperacillin/tazobactam 4 g/0.5 g three times daily and vancomycin 1.5 g twice daily and later changed to trimethoprim/sulphamethoxazole 160 mg/800 mg orally three times daily. Six weeks after admission, the patient could be discharged from the hospital. At follow-up the patient had only minor pain from the hip, but CRP was still not normal after 6 weeks of oral therapy; amoxicillin 1 g three times daily was therefore added to the treatment. The patient was treated with trimethoprim/sulphamethoxazole and amoxicillin orally for 6 months. At discontinuation of treatment, the patient’s CRP was 9.3 mg/L and his erythrocyte sedimentation rate (ESR) was 8 mm/h. The second-stage surgery was performed 10 months after the first-stage revision, and one aspiration of hip fluid and six tissue biopsies did not show any growth.

## Results

### Genome Sequencing of *P*. *avidum* Isolates

The *P*. *avidum* isolate from case 1 obtained in 2014 (designated *P*. *avidum* T14) and two *P*. *avidum* isolates from case 2 were selected for whole genome sequencing. For the latter, the initital isolate from 2013 (*P*. *avidum* T13) and the isolate obtained 18 months later (*P*. *avidum* T15) were both sequenced in order to see if these isolates represented the same clone. Illumina sequencing resulted in draft genomes consisting of 15, 9, and 14 contigs for *P*. *avidum* T13, T14, and T15, respectively. All three genomes had an identical G+C content (63.4%). The genome of *P*. *avidum* T14 is approximately 60 kb larger than the genomes of T13 and T15 (2,522,071 bp vs. 2,463,971 bp and 2,462,788 bp, respectively). This can be explained by the presence of a prophage in *P*. *avidum* T14 with high sequence similarity to prophage regions in the genomes of different species of *Corynebacterium* (*C*. *resistens*, *C*. *diphtheriae*, and *C*. *atypicum*). Previously published *P*. *avidum* genomes have displayed sizes slightly larger or comparable to *P*. *avidum* T14 (2,526,138 bp for *P*. *avidum* 44067 and 2,533,496 bp for *P*. *avidum* ATCC 25577). Using RAST [19], the number of identified coding sequences (CDS) is 2292, 2350, and 2288 for T13, T14 and T15, respectively. Each genome contains 49 RNAs. Interestingly, T13 and T15 are clonal. T15 is identical to T13, with the exception of the introduction of 331 single nucleotide polymorphisms (SNPs).

**Fig 3:**
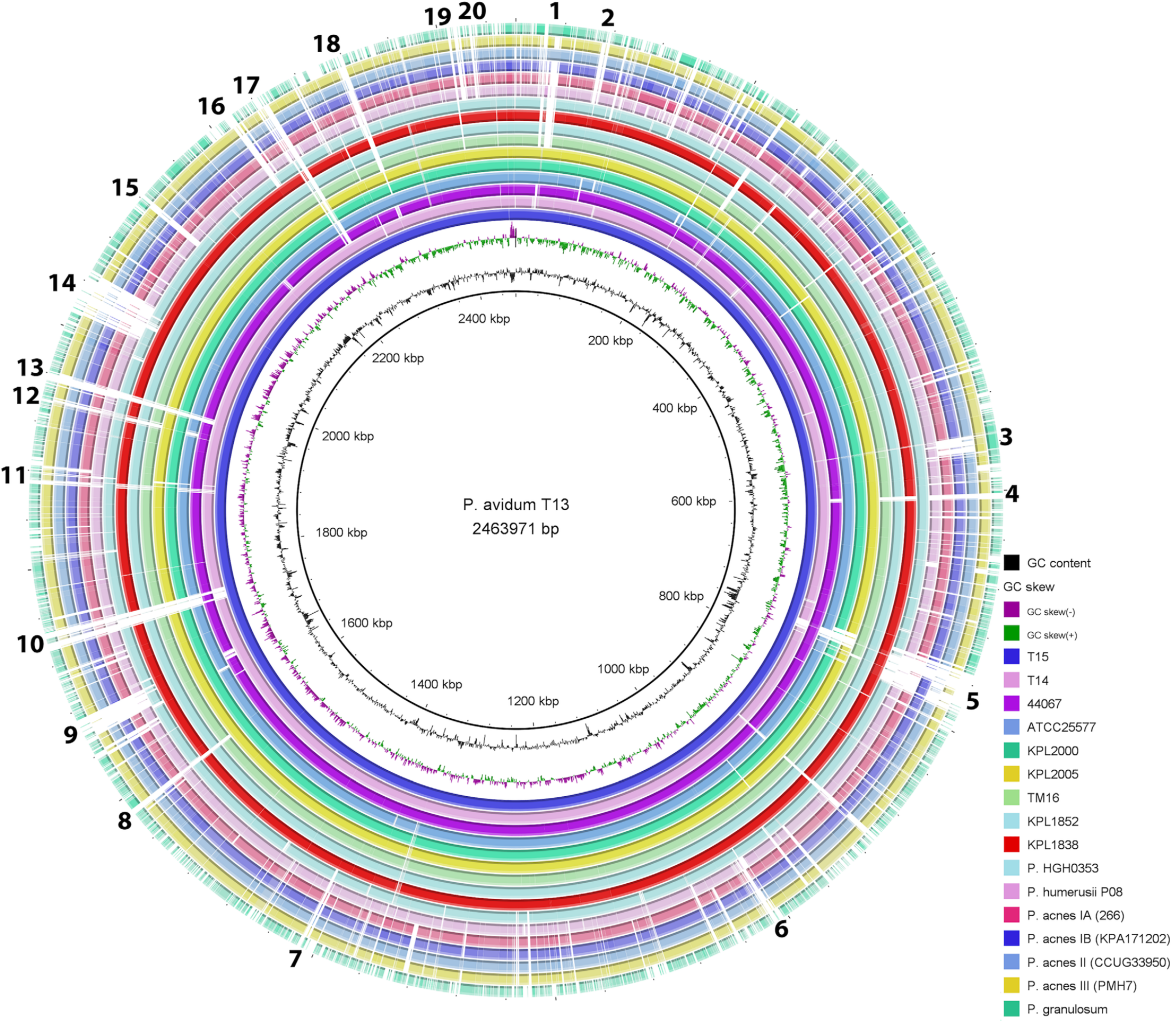
Genome comparison of *P*. *avidum* and other cutaneous Propionibacteria. *P*. *avidum* strain T13 was used as the reference genome. The most inner rings represent GC content and GC skew variation. Other *P*. *avidum* genomes (rings from inside to outside) are added according to their similarity to the reference genomes, i.e. T15 is most similar and HGH0353 is most dissimilar to T13. The comparison of *P*. *avidum* genomes with representative genomes *of P*. *acnes/P*. *humerusii/P*. *granulosum* shows overall genome synteny but also revealed P. avidum-specific genomic regions. These islands are numbered (1–20); gene content of the islands is shown in Table A1 in S1 File. Island 14 is the locus encoding EPS biosynthesis. The BRIG program was used to generate the figure.

In order to address the question about the presence and nature of species-specific traits, a detailed genome comparison of *P*. *avidum* and representative genomes of *P*. *acnes*, *P*. *humerusii* and *P*. *granulosum* was conducted (Fig 3). This analysis shows an overall genome synteny between cutaneous Propionibacteria. It also reveals *P*. *avidum* -specific sequences, i.e. genomic regions that are only present in *P*. *avidum* and lack in other cutaneous Propionibacteria (Fig 3 and Table A1-3 in S1 File.). One such region (island 14 in Fig 3) is a gene cluster encoding exopolysaccharide (EPS) synthesis; the EPS is likely to be responsible for strong adherence [15]. The 35 kbp EPS regions in strains T13 and T15 are identical on the nucleotide level, and 96% identical to the EPS region in strain T14. In addition to the EPS locus, other larger (> 5kb) genomic regions are found in *P*. *avidum* genomes that are absent from selected genomes of *P*. *acnes* (Fig 2 and Table A1 in S1 File.). Gene content comparison using ProteinOrtho [20] revealed 283 and 211 CDS specific for *P*. *avidum* and *P*. *acnes*, respectively (Table A2 and A3 in S1 File.). Diverse fitness, survival and defense functions are encoded in the *P*. *avidum*-specific regions, including a variety of transporters of the ABC-type predicted to be specific for the transport of sugars, amino acids (methionine, glutamine) and iron or hemin. Other loci harbor genes for a type I restriction-modification system and for arsenic resistance. Two toxin-antitoxin systems are found in one genomic locus, a HigA/HigB system and two genes with homology to the death-on-curing (DOC) system of some bacteriophages.

**Fig 4:**
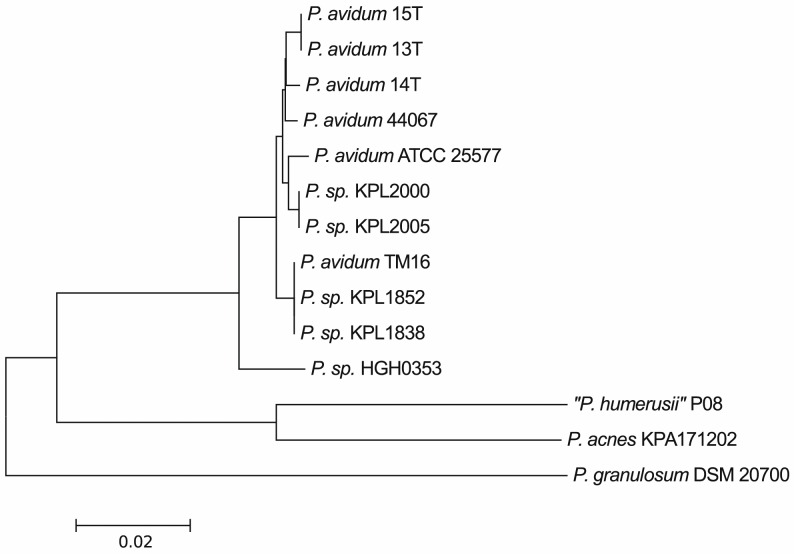
A phylogenetic analysis of sequenced *P*. *avidum* strains and their relation to other cutaneous Propionibacteria. The analysis is based on the shared core genome of the depicted organisms. T13, T14, and T15 cluster closely not only with each other, but also with strain 44067 (14), another clinical isolate from an abscess. Some strains (KPL2000, KPL2005, KPL1852, KPL1838) are currently not correctly assigned to *P*. *avidum* in the public databases. The population of *P*. *avidum* can be divided into three (phylogenetic) clusters: cluster A: T13, T14, T15, 44067; cluster B: ATCC25577, KPL2000, KPL2005, TM16; cluster C: KPL1852, KPL1838). Their gene content differences are shown in Table B1-3 in S2 File.

Several of the *P*. *avidum*-specific genomic regions seem to be acquired by horizontal gene transfer, including the EPS locus. They are flanked with tRNA genes, which are known to frequently serve as integration sites for mobile genetic elements. A phylogenetic analysis of the *P*. *avidum* strains sequenced so far showed that the strains T13, T14, and T15 cluster closely with each other and also with strain 44067 [21], a clinical isolate from an abscess (Fig 4). The phylogenetic analysis showed that three different clusters are distinguishable within the *P*. *avidum* population. Gene content analysis revealed 12, 29 and 72 cluster-specific CDS for cluster A (strains T13, T14, T15 and 44067), cluster B (strains ATCC25577, KPL2000 and KPL2005) and cluster C (strains KPL1852 and KPL1838), respectively (Table A1-3 in S2 File.). This shows that different types of *P*. *avidum* can be found on the human body.

## Discussion

These are, to our knowledge, the first reported cases of *P*. *avidum* as the etiological agent of deep periprosthetic infections. Both cases had a subacute onset of symptoms that developed during the first couple of weeks after primary hip arthroplasty. Case 1 saw a successful outcome following a DAIR [22] procedure (debridement, antibiotics, and implant retention), but in case 2 the DAIR failed. The patient was feeling well and the wound healed properly, but the infection remained dormant until a complete septic loosening of the acetabular component and loss of bone stock was present. The two patients were both obese and primarily operated with an anteriorly curved skin incision approach close to the skin crease of the groin. The most common surgical approaches to total hip arthroplasties are the posterolateral and the direct lateral approaches [23,24]. In these approaches, the skin incision is distant to the groin. However, there is a trend towards performing more arthroplasty surgery using anteriorly placed incisions [25,26] with or without mini-incision. The main advantage of anteriorly based approaches is that they are tissue sparring by avoiding cutting major muscles and reduces the dislocation rate [27–31]. Surgery with mini-incision technique is thought to reduce the tissue damage even more and promote faster recovery [32,33].

*Propionibacterium* spp. are the main constituents of the anaerobic microbiota of the skin, and are predominantly found in oily, sebaceous-rich areas. *P*. *acnes* are the most prevalent *Propionibacterium* spp. on human skin; it primarily colonizes sebaceous skin follicles. In contrast, *P*. *avidum* is often isolated from moist areas of the skin such as the nares, axilla, rectum, and groin [34]. Incisions closer to or in the groin area, such as the anteriorly curved skin incision approach, may result in an increased risk of contamination of the surgical wound area with skin commensals present in the moist milieu of the groin, and thus emergence of *P*. *avidum* as a cause of infections following total hip arthroplasty surgery.

Strains of *P*. *avidum* have been shown to be surrounded by an EPS-like structure [15]. This structure could play a role in adherence and biofilm formation and thus may contribute to pathogenicity and subsequently to the emergence of chronic foreign body infections. EPS production could also reduce the susceptibility of the strain to antibiotic treatment. The present study confirms that the gene cluster encoding EPS biosynthesis is–among the cutaneous propionibacterial species- unique to *P*. *avidum*; it was identified in all three newly sequenced isolates.

The putative biofilm forming ability of *P*. *avidum* is relevant regarding the optimal antimicrobial treatment of foreign body infections caused by *P*. *avidum*. The present isolates of the two cases showed low minimum inhibitory concentration (MIC) values against beta-lactam antibiotics such as penicillin G, and were susceptible to several additional antimicrobials with the exception of metronidazole. Thus, the MIC values do not differ from what has been reported for *P*. *acnes* isolated from implant-related infections [35].

In Sweden, cloxacillin is the drug of choice for prophylaxis during prosthetic joint surgery. However, since *P*. *acnes* has been recognized as an important etiological agent in shoulder PJIs [36]there is a discussion and also a trend today towards adding benzyl-penicillin to the routine prophylaxis for shoulder prosthetic surgery. The MIC values of benzyl-penicillin of *P*. *acnes* are in general very low. However, the MIC values for oxacillin is also low [37]. The timing of the routine prophylaxis is equally important issue [38]. Further studies to investigate if there should be a change in the antibiotic prophylaxis, e.g. by adding benzyl-penicillin, for patients undergoing total hip arthroplasty with an anterior skin incision approach is however warranted. Furthermore, to determine the MIC values of *Propionibacterium* spp. isolated from PJIs to beta-lactam antibiotics, since no current data are available [37].

The optimal antibiotics for the treatment of PJIs caused by *P*. *acnes* have not been fully determined. Both beta-lactam antibiotics [39,40] and rifampicin in combination with, for example, moxifloxacin have been proposed [7]. According to in vitro and in vivo animal experimental studies, rifampicin containing combinations of antimicrobial agents were favorable for eradication of *P*. *acnes* biofilms [41]. The fact that the *P*. *avidum* isolates obtained from the patients in the present case report harbored genes encoding EPS biosynthesis may be considered when deciding long-term treatment. The present *P*. *avidum* isolates were both fully susceptible to beta-lactam antibiotics, fluoroquinolones, and rifampicin. Case 1 was successfully treated with debridement and rifampicin in combination with clindamycin for approximately 1 month and then an additional 2 months of antimicrobial therapy with phenoxymethylpenicillin. The final outcome of case 2 was a two-stage exchange intervention of the hardware due to loosening, despite early debridement. In this case, antibiotic treatment with clindamycin was administered as monotherapy for about 6 weeks. At re-operation 18 months later the isolate was still fully susceptible to clindamycin.

The re-isolation of the same strain in case 2 underlines the persistence and long-term colonization capacity of *P*. *avidum*. The observed SNPs between the original strain and the strain isolated 18 months later might give interesting insights into the evolution of *P*. *avidum* during colonization of the human host.

Only very limited genomic and phenotypic information about the species *P*. *avidum* is currently available. Our data indicates that the *P*. *avidum* population can be dived in at least three different phylogenetic clusters. However, the number of sequenced strains is currently very low, making it difficult to evaluate if disease-associated strains of *P*. *avidum* can be distinguished phylogenetically from health-associated ones and whether disease-associated strains possess an elevated pathogenic potential.

## Conclusions

The two cases presented here indicate that *P*. *avidum* has a pathogenic potential and the ability to cause clinically relevant infections including abscess formation when foreign bodies, such as prosthetic joint components, are present. We recommend increased awareness for *Propionibacterium* spp. infection in hip arthroplasty if the skin incision is in close proximity to the groin or a deep skin crease. Consequently, obese patients might be at higher risk.

## Material and Methods

### Ethics Statement

The study material in this case report is bacterial isolates. Therefore, an ethical approval has not been obtained. However, written informed consent for publication has been obtained from both patients and personal identifiers has been removed to give confidentiality.

### Bacterial Cultivation

The three clinical *P*. *avidum* isolates T13, T14 and T15 obtained in this study were cultivated on Reinforced Clostridial Agar (Oxoid) plates for 3 days at 37°C under anaerobic conditions using the Gas-Pak™ system (Oxoid). For liquid cultures, brain heart infusion (BHI) broth (Sigma-Aldrich) was used and cultures were grown for 24 h at 37°C under anaerobic conditions.

### Building a *P*. *avidum* Core Genome Phylogenetic Tree

Genome sequences of publicly available *P*. *avidum* strains were used to build a phylogenetic tree based on all sequences shared with (one representative strain of) the species *P*. *acnes*, *P*. *granulosum* and *“P*. *humerusii”*. Genomes were downloaded from NCBI Genome and WGS databases, with the exception of strains T13, T14 and T15 that were sequenced for this study. In total, 11 *P*. *avidum* genomes were used. The core genome was identified by slicing the genome sequence of strain *P*. *avidum* 44067 into fragments of 200 bp which were used as query sequences in blastn (Blast+) [42] to extract homologous sequences from the other genomes. Default parameters of blastn were used together with a 65% cut-off on coverage. Homologous sequences of each fragment were aligned using Muscle [43] and subsequently all fragments were concatenated into one sequence (397,681 bp) per strain using a python script. The core genome phylogenetic tree was built in Mega v6 [44] using the minimal-evolution algorithm with complete deletion.

### Genome Sequencing

Genomic DNA from cultures of *P*. *avidum* T13, T14 and T15 was isolated using the MasterPure Gram-positive DNA Purification Kit (EpiCentre MGP04100) according to the manufacturer´s instructions. The purity and quality of the gDNA were assessed on a 1% agarose gel and with a nanodrop apparatus (Thermo Scientific, Wilmington USA). Extracted DNA was used to prepare Nextera XT shotgun libraries for the Genome Analyzer II (Illumina, San Diego, CA, USA) with a 112-bp paired-end sequencing run. Libraries were prepared according to the manufacturer protocol at the Göttingen Genomics Laboratory, Germany. Raw reads were quality controlled with FastQC v0.11.2 (http://www.bioinformatics.bbsrc.ac.uk/projects/fastqc) and subsequently trimmed using Trimmomatic 0.32 (http://www.usadellab.org/cms/?page=trimmomatic) to remove sequences with quality scores lower than 20 (Illumina 1.9 encoding) and remaining adaptor sequences. De novo assembly was done using the SPAdes v3.5 software [45]. Key features of the assembly are as follows: T13: 15 contigs with a total of 2,463,971 bp; the final assembly had a N50 value of 486,392 bp and a N90 value of 89,725 bp with a 50.4-fold coverage (excluding 16S rRNA operons). T14: 9 contigs with a total of 2,522,071 bp; the final assembly had a N50 value of 802,850 bp and a N90 value of 278,150 bp with a 53-fold coverage (excluding 16S rRNA operons). T15: 14 contigs with a total of 2,462,788 bp; the final assembly had a N50 value of 289,009 bp and a N90 value of 97,503 bp with a 21-fold coverage (excluding 16S rRNA operons). The GenBank accession numbers of the draft genome sequences are LLJH00000000 (T13), LLJI00000000 (T14) and LLJJ00000000 (T15).

### Gene Prediction, Annotation and Comparative Genome Analysis

For *P*. *avidum* strains T13, T14 and T15 open reading frames (ORFs) and tRNAs were identified and annotated using the RAST server [19]. For comparative genome analysis, the programs ACT (http://www.sanger.ac.uk/science/tools/artemis-comparison-tool-act) and BRIG [46] were used, resulting in the identifciation of *P*. *avidum* and *P*. *acnes*-specific islands, respectively. The following genomes of Propionibacteria were taken from GenBank to build a BRIG genome comparison: *P*. *avidum*: 44067 [BioProject: PRJNA157751], ATCC 25577 [BioProject: PRJNA64741], and TM16 [BioProject: PRJNA189036]; other *P*. *avidum* genomes from strains that are currently (insufficiently) designated as *Propionibacterium* sp.: KPL2005 [BioProject: PRJNA170373], KPL2000 [BioProject: PRJNA170369], KPL1852 [BioProject: PRJNA169465], and KPL1838 [BioProject: PRJNA169461]; *P*. *humerusii*: P08 [BioProject: PRJNA64551]; *P*. *acnes*: 266 [BioProject: PRJNA56091], KPA171202 [BioProject: PRJNA12460], and PMH7 [BioProject: PRJNA292394].

### Gene Content Analysis

In order to compare the gene content between *P*. *avidum* and *P*. *acnes* genomes and within the *P*. *avidum* isolates we extracted all annotated proteins (CDS) with the script cds_extractor.pl (v0.7.1) (https://github.com/aleimba/bac-genomics-scripts). The software Proteinortho (v5.11) was used to identify orthologs and cluster the proteins into orthologous groups (OGs) with a 70% identity and coverage cutoff and options “-selfblast” and “-singles” for BLASTP+ [20,42]. Additionally, BLASTP+ was run with an E-value of 1 x 10^−5^, a final Smith-Waterman alignment (option “-use_sw_tback”) and without SEG filtering (option “-seg no”) [47].

The script po2group_stats.pl (v0.1.2) (https://github.com/aleimba/bac-genomics-scripts) was used to identify species-specific OGs between *P*. *avidum* (44067, T13, T14, T15, ATCC 25577, KPL2000, KPL2005, KPL1838, and KPL1852) and *P*. *acnes* genomes (266 (IA), 12.1.R1 (IA), KPA171202 (IB), CCUG33950 (II), and PMH7 (III)). Additionally, we sought to identify OGs specific for different subgroups of *P*. *avidum* genomes based on the phylogeny (Fig 4). Again po2group_stats.pl was used to identify these group-specific OGs between *P*.*avidum* subcluster A (strains 44067, T13, T14 and T15), subcluster B (strains ATCC25577, KPL2000 and KPL2005), and subcluster C (strains KPL1852 and KPL1838).

## Supporting Information

S1 File P. *avidum*-Specific genomic regions (>5kb) that are absent from selected genomes of *P*.*acnes* types 1A,1B,1C.
(XLSX)

S2 File CDS specific to subcluster A, B and C of *P*. *avidum* (compared to the other subclusters A, B and C).
(XLSX)

## References

[1] Learmonth ID, Young C, Rorabeck C. The operation of the century: total hip replacement. Lancet. 2007;370(9597):1508–19. 17964352 10.1016/S0140-6736(07)60457-7

[2] Tande AJ, Patel R. Prosthetic joint infection. Clin Microbiol Rev. 2014;27(2):302–45. 10.1128/CMR.00111-13 24696437 PMC3993098

[3] Perry AL, Lambert PA. Propionibacterium acnes. Lett Appl Microbiol. 2006;42(3):185–8. 16478502 10.1111/j.1472-765X.2006.01866.x

[4] Perry A, Lambert P. Propionibacterium acnes: infection beyond the skin. Expert Rev Anti Infect Ther. 2011;9(12):1149–56. 10.1586/eri.11.137 22114965

[5] Zimmerli W, Trampuz A, Ochsner PE. Prosthetic-joint infections. N Engl J Med. 2004;351(16):1645–54. 15483283 10.1056/NEJMra040181

[6] Zimmerli W, Moser C. Pathogenesis and treatment concepts of orthopaedic biofilm infections. FEMS Immunol Med Microbiol. 2012;65(2):158–68. 10.1111/j.1574-695X.2012.00938.x 22309166

[7] Lutz MF, Berthelot P, Fresard A, Cazorla C, Carricajo A, Vautrin AC, et al Arthroplastic and osteosynthetic infections due to Propionibacterium acnes: a retrospective study of 52 cases, 1995–2002. Eur J Clin Microbiol Infect Dis. 2005;24(11):739–44. 16328558 10.1007/s10096-005-0040-8

[8] Zeller V, Ghorbani A, Strady C, Leonard P, Mamoudy P, Desplaces N. Propionibacterium acnes: an agent of prosthetic joint infection and colonization. J Infect. 2007;55(2):119–24. 17418419 10.1016/j.jinf.2007.02.006

[9] Piper KE, Jacobson MJ, Cofield RH, Sperling JW, Sanchez-Sotelo J, Osmon DR, et al Microbiologic diagnosis of prosthetic shoulder infection by use of implant sonication. J Clin Microbiol. 2009;47(6):1878–84. 10.1128/JCM.01686-08 19261785 PMC2691098

[10] Nystrom LM, Wyatt CM, Noiseux NO. Arthroplasty infection by Priopionibacterium granulosum treated with reimplantation despite ongoing purulent-appearing fluid collection. J Arthroplasty. 2013;28(1):198 e5–8. 10.1016/j.arth.2012.03.004 22552222

[11] Panagea S, Corkill JE, Hershman MJ, Parry CM. Breast abscess caused by Propionibacterium avidum following breast reduction surgery: case report and review of the literature. J Infect. 2005;51(5):e253–5. 15908006 10.1016/j.jinf.2005.04.005

[12] Vohra A, Saiz E, Chan J, Castro J, Amaro R, Barkin J. Splenic abscess caused by Propionibacterium avidum as a complication of cardiac catheterization. Clin Infect Dis. 1998;26(3):770–1. 9524868 10.1086/517127

[13] Janvier F, Delacour H, Larreche S, Abdalla S, Aubert P, Merens A. Abdominal wall and intra-peritoneal abscess by Propionibacterium avidum as a complication of abdominal parietoplasty. Pathol Biol (Paris). 2013;61(5):223–5. 23415274 10.1016/j.patbio.2013.01.008

[14] Million M, Roux F, Cohen Solal J, Breville P, Desplaces N, Barthas J, et al Septic arthritis of the hip with Propionibacterium avidum bacteremia after intraarticular treatment for hip osteoarthritis. Joint Bone Spine. 2008;75(3):356–8. 10.1016/j.jbspin.2007.06.012 18329936

[15] Mak TN, Schmid M, Brzuszkiewicz E, Zeng G, Meyer R, Sfanos KS, et al Comparative genomics reveals distinct host-interacting traits of three major human-associated propionibacteria. BMC Genomics. 2013;14:640 10.1186/1471-2164-14-640 24053623 PMC3848858

[16] Bertin KC, Rottinger H. Anterolateral mini-incision hip replacement surgery: a modified Watson-Jones approach. Clin Orthop Relat Res. 2004(429):248–55. 15577495

[17] Muller M, Tohtz S, Springer I, Dewey M, Perka C. Randomized controlled trial of abductor muscle damage in relation to the surgical approach for primary total hip replacement: minimally invasive anterolateral versus modified direct lateral approach. Arch Orthop Trauma Surg. 2011;131(2):179–89. 10.1007/s00402-010-1117-0 20490520

[18] Paprosky WG, Perona PG, Lawrence JM. Acetabular defect classification and surgical reconstruction in revision arthroplasty. A 6-year follow-up evaluation. J Arthroplasty. 1994;9(1):33–44. 8163974 10.1016/0883-5403(94)90135-x

[19] Aziz RK, Bartels D, Best AA, DeJongh M, Disz T, Edwards RA, et al The RAST Server: rapid annotations using subsystems technology. BMC Genomics. 2008;9:75 10.1186/1471-2164-9-75 18261238 PMC2265698

[20] Lechner M, Findeiss S, Steiner L, Marz M, Stadler PF, Prohaska SJ. Proteinortho: detection of (co-)orthologs in large-scale analysis. BMC Bioinformatics. 2011;12:124 10.1186/1471-2105-12-124 21526987 PMC3114741

[21] Ordogh L, Hunyadkurti J, Voros A, Horvath B, Szucs A, Urban E, et al Complete Genome Sequence of Propionibacterium avidum Strain 44067, Isolated from a Human Skin Abscess. Genome announcements. 2013;1(3). 10.1128/genomeA.00337-13 PMC3675521 23792747

[22] Parvizi J, Zmistowski B, Adeli B. Periprosthetic joint infection: treatment options. Orthopedics. 2010;33(9):659 10.3928/01477447-20100722-42 20839679

[23] Chechik O, Khashan M, Lador R, Salai M, Amar E. Surgical approach and prosthesis fixation in hip arthroplasty world wide. Arch Orthop Trauma Surg. 2013;133(11):1595–600. 10.1007/s00402-013-1828-0 23912418

[24] Petis S, Howard JL, Lanting BL, Vasarhelyi EM. Surgical approach in primary total hip arthroplasty: anatomy, technique and clinical outcomes. Can J Surg. 2015;58(2):128–39. 25799249 10.1503/cjs.007214 PMC4373995

[25] Leunig M, Faas M, von Knoch F, Naal FD. Skin crease 'bikini' incision for anterior approach total hip arthroplasty: surgical technique and preliminary results. Clin Orthop Relat Res. 2013;471(7):2245–52. 10.1007/s11999-013-2806-0 23412730 PMC3676627

[26] Alecci V, Valente M, Crucil M, Minerva M, Pellegrino CM, Sabbadini DD. Comparison of primary total hip replacements performed with a direct anterior approach versus the standard lateral approach: perioperative findings. J Orthop Traumatol. 2011;12(3):123–9. 10.1007/s10195-011-0144-0 21748384 PMC3163771

[27] Woo RY, Morrey BF. Dislocations after total hip arthroplasty. J Bone Joint Surg Am. 1982;64(9):1295–306. 7142237

[28] Masonis JL, Bourne RB. Surgical approach, abductor function, and total hip arthroplasty dislocation. Clin Orthop Relat Res. 2002(405):46–53. 12461355 10.1097/00003086-200212000-00006

[29] Lindgren V, Garellick G, Karrholm J, Wretenberg P. The type of surgical approach influences the risk of revision in total hip arthroplasty: a study from the Swedish Hip Arthroplasty Register of 90,662 total hipreplacements with 3 different cemented prostheses. Acta Orthop. 2012;83(6):559–65. 10.3109/17453674.2012.742394 23116440 PMC3555460

[30] Tsukada S, Wakui M. Lower Dislocation Rate Following Total Hip Arthroplasty via Direct Anterior Approach than via Posterior Approach: Five-Year-Average Follow-Up Results. Open Orthop J. 2015;9:157–62. 10.2174/1874325001509010157 26157532 PMC4483535

[31] Connolly KP, Kamath AF. Direct anterior total hip arthroplasty: Comparative outcomes and contemporary results. World Journal of Orthopedics. 2016;7(2):94–101. 10.5312/wjo.v7.i2.94 26925380 PMC4757663

[32] Bergin PF, Doppelt JD, Kephart CJ, Benke MT, Graeter JH, Holmes AS, et al Comparison of minimally invasive direct anterior versus posterior total hip arthroplasty based on inflammation and muscle damage markers. J Bone Joint Surg Am. 2011;93(15):1392–8. 10.2106/JBJS.J.00557 21915544 PMC3143583

[33] Xu CP, Li X, Song JQ, Cui Z, Yu B. Mini-Incision versus Standard Incision Total Hip Arthroplasty Regarding Surgical Outcomes: A Systematic Review and Meta-Analysis of Randomized Controlled Trials. PLoS One. 2013;8(11). 10.1371/journal.pone.0080021 PMC3827164 24265792

[34] McGinley KJ, Webster GF, Leyden JJ. Regional variations of cutaneous propionibacteria. Appl Environ Microbiol. 1978;35(1):62–6. 623473 10.1128/aem.35.1.62-66.1978 PMC242779

[35] Khassebaf J, Hellmark B, Davidsson S, Unemo M, Nilsdotter-Augustinsson A, Soderquist B. Antibiotic susceptibility of Propionibacterium acnes isolated from orthopaedic implant-associated infections. Anaerobe. 2015;32:57–62. 10.1016/j.anaerobe.2014.12.006 25541476

[36] Wang B, Jessamine P, Desjardins M, Toye B, Ramotar K. Direct mecA polymerase chain reaction testing of blood culture bottles growing Gram-positive cocci and the clinical potential in optimizing antibiotic therapy for staphylococcal bacteremia. Diagn Microbiol Infect Dis. 2013;75(1):37–41. 10.1016/j.diagmicrobio.2012.09.014 23102997

[37] Wang WL, Everett ED, Johnson M, Dean E. Susceptibility of Propionibacterium acnes to seventeen antibiotics. Antimicrob Agents Chemother. 1977;11(1):171–3. 836012 10.1128/aac.11.1.171 PMC351938

[38] Stefansdottir A, Robertsson O, A WD, Kiernan S, Gustafson P, Lidgren L. Inadequate timing of prophylactic antibiotics in orthopedic surgery. We can do better. Acta Orthop. 2009;80(6):633–8. 10.3109/17453670903316868 19995312 PMC2823303

[39] Bayston R, Nuradeen B, Ashraf W, Freeman BJ. Antibiotics for the eradication of Propionibacterium acnes biofilms in surgical infection. J Antimicrob Chemother. 2007;60(6):1298–301. 17959732 10.1093/jac/dkm408

[40] Sendi P, Zimmerli W. Antimicrobial treatment concepts for orthopaedic device-related infection. Clin Microbiol Infect. 2012;18(12):1176–84. 10.1111/1469-0691.12003 23046277

[41] Furustrand Tafin U, Corvec S, Betrisey B, Zimmerli W, Trampuz A. Role of rifampin against Propionibacterium acnes biofilm in vitro and in an experimental foreign-body infection model. Antimicrob Agents Chemother. 2012;56(4):1885–91. 10.1128/AAC.05552-11 22252806 PMC3318339

[42] Camacho C, Coulouris G, Avagyan V, Ma N, Papadopoulos J, Bealer K, et al BLAST+: architecture and applications. BMC Bioinformatics. 2009;10:421 10.1186/1471-2105-10-421 20003500 PMC2803857

[43] Edgar RC. MUSCLE: multiple sequence alignment with high accuracy and high throughput. Nucleic Acids Res. 2004;32(5):1792–7. 15034147 10.1093/nar/gkh340 PMC390337

[44] Tamura K, Stecher G, Peterson D, Filipski A, Kumar S. MEGA6: Molecular Evolutionary Genetics Analysis version 6.0. Mol Biol Evol. 2013;30(12):2725–9. 10.1093/molbev/mst197 24132122 PMC3840312

[45] Bankevich A, Nurk S, Antipov D, Gurevich AA, Dvorkin M, Kulikov AS, et al SPAdes: a new genome assembly algorithm and its applications to single-cell sequencing. J Comput Biol. 2012;19(5):455–77. 10.1089/cmb.2012.0021 22506599 PMC3342519

[46] Alikhan NF, Petty NK, Ben Zakour NL, Beatson SA. BLAST Ring Image Generator (BRIG): simple prokaryote genome comparisons. BMC Genomics. 2011;12:402 10.1186/1471-2164-12-402 21824423 PMC3163573

[47] Moreno-Hagelsieb G, Latimer K. Choosing BLAST options for better detection of orthologs as reciprocal best hits. Bioinformatics. 2008;24(3):319–24. 18042555 10.1093/bioinformatics/btm585

